# Platelet Distribution Width and Mortality in Hemodialysis Patients

**DOI:** 10.1155/2021/6633845

**Published:** 2021-03-16

**Authors:** Wang Ruiyan, Xu Bin, Dong Jianhua, Zhou Lei, Gong Dehua, Zheng Tang

**Affiliations:** ^1^Jinling Hospital Department of Nephrology, Nanjing Medical University, Nanjing, China; ^2^Department of Nephrology, Shanghai Fourth People's Hospital Affiliated to Tongji University School of Medicine, Shanghai, China; ^3^Jinling Hospital Research Institute of Kidney Disease, Nanjing University School of Medicine, Nanjing, China

## Abstract

**Objectives:**

The association between platelet distribution width (PDW) and mortality in hemodialysis (HD) patients has received little attention.

**Methods:**

We retrospectively enrolled HD patients in a single center from January 1, 2008, to December 30, 2011. The primary and secondary endpoints were all-cause and cardiovascular mortality, respectively. The association between PDW and mortality was estimated by Cox regression model.

**Results:**

Of 496 patients, the mean age was 52.5 ± 16.6 years, and the Charlson comorbidity index was 4.39 ± 1.71. During the follow-up period of 48.8 ± 6.7 months, 145 patients (29.2%) died, including 74 (14.9%) cardiovascular deaths. 258 (52.0%) with PDW < 16.31% were in the low group and 238 (48.0%) in those with PDW ≥ 16.31% according to cut-off for all-cause mortality by receiving-operator characteristics. After adjusting for confounding factors, high PDW values were independently associated with higher risk of all-cause (hazards ratio (HR) = 1.49, 95% confidence interval (CI) 1.15–6.82) and cardiovascular deaths (HR = 2.26, 95% CI 1.44–3.63) in HD patients. When comparing with quartile 1 of PDW, quartile 4 of PDW was independently associated with a higher risk of all-cause (HR = 1.59, 95% CI 1.18–5.30) and cardiovascular deaths (HR = 2.71, 95% CI 1.49–3.76) in HD patients.

**Conclusions:**

Baseline PDW was independently associated with all-cause and cardiovascular mortality in HD patients.

## 1. Introduction

According to the 2015 annual data report of kidney disease surveillance network in China, hemodialysis, which is about 402.18 per million in prevalence rate and 553,000 patients when quantified, is the primary renal replacement therapy for end-stage renal disease (ESRD) patients [1]. Patients on maintenance hemodialysis (HD) exhibit a high mortality, mainly due to cardiovascular events [2]. Among patients with ESRD, high concentrations of uremic toxins, chronic inflammation state, and broken hemostasis impaired platelet activation, including adhesiveness, aggregation, and release function, lead to the worse prognosis because of both bleeding or thrombosis events [3]. Besides extrinsic anticoagulation, blood-membrane reaction during hemodialysis may aggravate and amplified this process [4]. An increasing body of evidence suggests that platelet distribution width (PDW), an indicator representing the heterogeneity of platelet size, is a potent marker of platelet activity [5–8]. Several studies show that higher PDW levels are associated with risk factors of cardiovascular disease such as hyperuricemia, diabetes, and metabolic syndrome [9–11]. Studies also show that a lower PDW was related to a poor outcome in acute ischemic stroke patients on intravenous thrombolysis, mild cognitive impairment, and Alzheimer's disease [12, 13].

A previous study found that the mean values of PDW in HD patients were higher than those of healthy volunteers. [14] However, the association between PDW values and the prognosis of HD patients has received little attention. Therefore, we aimed to evaluate the association between PDW values and mortality in HD patients.

## 2. Methods

### 2.1. Study Population

We performed a retrospective cohort study of HD patients (dialysis vintage was ≥ three months) between January 1, 2008, and December 30, 2011, in the blood purification center of the National Clinical Research Center of Kidney Disease in Jinglin Hospital. We excluded patients according to the following criteria: infections that needed antibiotic therapy, hematological diseases, immunosuppressant drugs or steroids therapy, antiplatelet therapy, or carcinomas. This study was conducted according to the principles expressed in the Declaration of Helsinki. The Ethics Committees of Jinglin Hospital approved the protocol of this study and waived the need for written informed consent because the data were analyzed anonymously.

Baseline variables included age, sex, Charlson comorbidity index (CCI), dialysis vintage, dry weight, body mass index (BMI), Kt/V, dialysis access, access thrombosis, and blood pressure. Laboratory indexes, including creatinine, albumin, high-sensitive C-reactive protein (hs-CRP), hemoglobin, platelet count, mean platelet volume (MPV), and PDW, were obtained from the first month of patients' enrollment.

The primary endpoint was all-cause death, and the secondary endpoint was cardiovascular death. Death certification was obtained from the mortality records of our center. Cardiovascular death was judged when death was caused by myocardial infarction, sudden cardiac death, heart failure, arrhythmia, cardiogenic shock, and stroke [15]. The comorbidity score was determined according to the CCI, which is one of the most commonly used comorbidity models. Dry weight was defined as the lowest weight a patient can tolerate without the presence of symptoms or hypotension [16]. Body mass index (BMI) before dialysis was calculated by dividing the dry weight in kilograms by height in meters squared. Kt/V was the clearance of urea multiplied by dialysis duration and normalized for urea distribution volume [17]. Access thrombosis defined as access (autologous arteriovenous fistula or catheter) continued anomaly due to clot, which could be detected by any physical examination, blood flow measurement, or static venous pressure.

### 2.2. Statistical Analysis

Receiving-operator characteristics (ROC) curves were applied to find a PDW cut-off value for predicting all-cause mortality. Participants were divided into two groups according to the PDW cut-off value of ROC. The results are presented as frequency and percentage for categorical variables, the mean and standard deviation (SD) for the continuous variables of the normal distribution, and the median and interquartile range for the nonnormal distribution parameters. Comparison between two groups proceeded through chi-square tests for categorical variables, unpaired *t-test* for continuous variables of the normal distribution, and nonparametric test for nonnormal distribution parameters. Logistic regression analyses were conducted to evaluate the association between baseline variables and high PDW (PDW ≥ 16.31%). Variables with *P* < 0.05 in the univariate analysis were included in a multivariate-adjusted model. Cumulative survival was estimated by Kaplan–Meier curves, and the difference between survival curves was compared through the log-rank test. The association between the PDW values and all-cause and cardiovascular mortality was estimated by the multivariable-adjusted Cox regression analysis. Unadjusted associations were first examined, followed by adjustments for age, sex, and CCI. Next, BMI, creatinine, albumin, hs-CRP, and MVP were added to examine whether the association of the PDW with all-cause and cardiovascular mortality was independent of confounding factors. Furthermore, patients with continuous PDW were classified into quartiles: quartile 1 < 10.53, quartile 2 = 10.53–14.67, quartile 3 = 14.68–18.86, and quartile 4 > 18.86. The association of the quartiles of PDW and all-cause and CVD mortality was further analyzed with the Cox regression models. The results of the Cox analysis were presented as the hazard ratio (HR) and the 95% CI. Data were analyzed by SPSS version 21.0 for Windows. *P* < 0.05 was considered statistically significant.

## 3. Results

### 3.1. Baseline Characteristics

**Figure 1:**
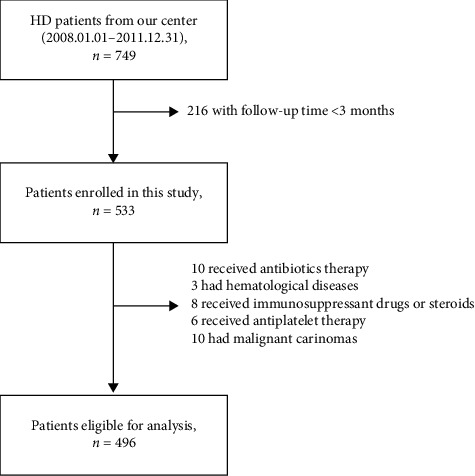
Flow chart showing the process of patients' enrollment.

A total of 749 patients received HD in this blood purification center. 215 of them received less than three months of HD. Thirty-eight patients were excluded due to different reasons: 10 patients received antibiotics because of infections, three had hematological diseases, eight received immunosuppressant drugs or steroids, six received antiplatelet therapy, and 10 had malignant carcinomas. The remaining 496 patients were included in the analysis (Figure 1).

The mean values of PDW were 14.37 ± 1.63%. According to the ROC curve analysis, a PDW cut-off value of 16.31% was obtained (area under the curve = 0.761, 95% CI = 0.690–0.831, *P*=0.037), which had 85% sensitivity and 77% specificity for differentiating the patients with a high risk of all-cause mortality. Patients were divided into two groups (PDW < 16.31% and PDW ≥ 16.31%). The baseline demographic characteristics and variables are summarized in Table 1.

The mean age was 52.5 ± 16.6 years, and 59.1% were male. Patients with higher PDW were likely to be male (*P* < 0.001) and had higher BMI (*P*=0.049), hs-CRP (*P* < 0.001), creatinine (*P*=0.005), and MPV (*P*=0.022) and had lower albumin values (*P*=0.033).

### 3.2. Association between Baseline Variables and High PDW Using Logistic Analysis

The prevalence of high PDW (PDW ≥ 16.31%) was 47.9% in the cohort study. Univariate logistic analysis found that age (*P* < 0.001), female sex (*P* < 0.001), CCI (*P* < 0.001), and albumin (*P*=0.019) were associated with high PDW. Multivariate logistic analysis showed that age (*P* < 0.001) and CCI (*P* < 0.001) were independently associated with the high PDW.

### 3.3. Association between PDW and Mortality

**Figure 2:**
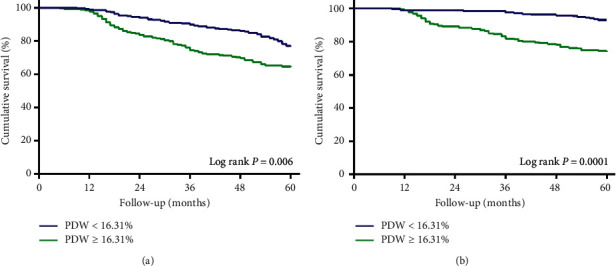
Cumulative all-cause and cardiovascular mortality in the PDW ≥ 16.31% or PDW < 16.31% groups. CVD, cardiovascular disease; PDW, platelet distribution width. (a) All-cause mortality. (b) CVD mortality.

A total of 16 patients had transferred to other hospitals, and six patients had received renal transplantations by the end of the study. No patients were lost to follow-up. There were 145 patients (29.2%) died, including 74 (51.0%) cardiovascular deaths. All-cause mortality and cardiovascular mortality in patients with higher levels of PDW were 36.6% and 19.3% and 22.5% and 10.9% in patients with lower levels of PDW. Kaplan–Meier analysis showed that cumulative all-cause mortality and cardiovascular mortality in the PDW ≥ 16.31% group were significantly higher than those in the PDW < 16.31% group (log-rank test: *P*=0.006 and *P*=0.0001, respectively, Figure 2).

Crude analysis showed that PDW ≥ 16.31% (PDW < 16.31% as a reference) was associated with higher risks of all-cause and cardiovascular deaths (HR = 1.53, 95% CI 1.33–5.92, *P*=0.001, and HR = 2.51, 95% CI 1.53–3.59, *P*=0.002, Table 2, Model 1). Multivariable analysis showed that PDW ≥ 16.31% was independently associated with higher risks of all-cause and cardiovascular deaths (HR = 1.49, 95% CI 1.15–6.82, *P*=0.007, and HR = 2.26, 95% CI 1.44–3.63, *P*=0.011, Table 2, Model 3).

Besides, patients with PDW quartile 4 had 1.59 and 2.71 times of all-cause and CVD mortality as compared with those with PDW quartile 1, respectively, when adjusting for confounding factors.

## 4. Discussion

In this retrospective cohort of HD patients, we observed that higher PDW levels were independently associated with a high risk of all-cause and cardiovascular deaths. In addition, we further found that there was a dose-dependent relationship between PDW at baseline and mortality in HD patients.

Platelets play a crucial role in maintaining vascular integrity, and hemostasis and activated platelet also aggregate at the site of atherosclerotic plaque rupture, stimulate thrombus formation, and promote atherothrombotic disease. Besides, platelets also take part in sterile inflammation and response to pathogens [18]. Given that dialysis patients are known to be chronically inflamed, platelet dysfunction is common in HD patients. Atherosclerosis is an inflammatory condition, and it seems that the abnormalities in platelets contribute to the access events and cardiovascular events of these HD patients [19, 20]. In our patients, 23.2% of patients had vascular access thrombosis, and 14.9% died from cardiovascular events, which accounted for 51.0% of all-cause mortality. This result is similar to previous research that cardiovascular disease accounts for 40% to 50% of deaths in patients with ESRD, which suggested that platelet activity may be related to the prognosis of HD patients [21].

Platelet counts, MPV, and PDW are indexes obtained quickly, and conveniently, the counts of platelets reflected the number of platelets, which are highly susceptible to the external environment like the usage of drugs. The MPV reflects the volume of platelets and the production of new platelets from bone marrow [22]. Recent evidence also showed that abnormal MPV is linked to diabetes, cardiovascular disease, and autoimmune disorders, as well as PDW [23, 24]. However, the MPV reference values are varied among different laboratories [22, 25]. Furthermore, MPV presents noticeable differences among different sexes, ages, and populations, as well as platelet counts, which may be due to genetic variations [26]. PDW is just an index reflecting the variation of platelet sizes independent of platelet counts and MPV [27]. It has been shown that a higher value of PDW presence a higher production of larger reticulated platelets, which have larger and more metabolical activity [28, 29]. Thus, PDW can be recognized as a sign of inflammation and coagulation disease. A PDW cut-off value of 16.45% (AUC = 0.870) could predict persistent organ failure in acute pancreatitis [30]. In addition, PDW ≥ 13.65% for predicting systemic lupus erythematosus (SLE) active stage and PDW ≥ 11.85% for SLE diagnosis was taken to grouping, which reported that PDW was positively correlated with SLE disease activity index and erythrocyte sedimentation rate, and the two indexes are commonly used in inflammatory diseases [31]. A cohort of 254 patients with intermediate (50%–70%) carotid artery stenosis were observed that PDW ≥ 14.3% were independent predictors of developing symptomatic carotid artery stenosis [6]. According to PDW values, patients classified into quartiles were applied in patients undergoing percutaneous coronary interventions [32]. In this study, the mean PDW values were 13.4 ± 2.5% and independently associated with a high risk of major adverse cardiovascular events. A large cohort of 31751 Chinese middle-aged and older people from the Dongfeng-Tongji cohort study showed that lower PDW values (PDW < 13.2%) were significantly related to lower risk of cardiovascular diseases compared with participants with 13.2% ≤ PDW ≤ 18.1%, after a median follow-up of 5.9 years [33]. Another study reported that PDW showed a significant increase from the first to the third trimester of pregnancy [8]. However, it was worth noting that a large sample size of 1882 patients undergoing coronary angiography found PDW was not related to the extent of coronary artery disease and carotid intima-media thickness. [28] Thus, the association between PDW and cardiovascular disease remained inconsistent, and the definition of high PDW was also inconsistent. Besides, the association between PDW and mortality remained unclear. In the present study, the mean values of PDW in the present study were 14.37 ± 1.63%, which were inconsistent with those in the previous studies. A previous study reported that PDW values of HD patients were higher than healthy people [14]. High PDW in our findings was defined according to the population with difference disease based on previous studies in this area. We found that older age and higher CCI were independently associated with the high PDW in HD patients in our study. To evaluate the association between PDW and all-cause and cardiovascular mortality, we divided eligible HD patients into the high PDW group and low PDW group according to the cut-off of a PDW value of 16.31%, which was analyzed by ROC curve (AUC = 0.661, sensitivity: 90%, and specificity: 47%). We found that higher PDW values may be an independent predictor for all-cause and cardiovascular mortality in HD patients, even after adjusting for confounding factors. Thus, as a common, readily available, and inexpensive biomarker, PDW could be a promising predictor to identify HD patients at high risk for all-cause and cardiovascular mortality. Further study should be conducted to evaluate whether the prognosis of HD patients may be improved by the management of PDW.

Several limitations should be mentioned in the present study. First, a retrospective study allows us to establish associations but not causal relationships. It was impossible for us to adjust all factors for all-cause and cardiovascular mortality, and the effect of residual confounding cannot be eliminated completely. Nonetheless, we adjusted for significant risk factors for all-cause and cardiovascular mortality. Second, all patients were from a single center in Nanjing, and the sample size is small, which means our study may lack generalization to other centers or other regions. Third, we only evaluated baseline variables rather than changes over time in all-cause and cardiovascular mortality. Finally, because HD patients were all Chinese in the present study, the results may not apply to other ethnic HD patients.

In conclusion, we found that PDW at baseline was independently associated with all-cause and cardiovascular deaths in HD patients. As a common, readily available, and inexpensive biomarker, PDW could be a promising parameter to identify HD patients at high risk for all-cause and cardiovascular mortality.

## Figures and Tables

**Table 1:** Baseline characteristics and laboratory parameters stratified by PDW.

	Cohort, *n* = 496	PDW < 16.31%, *n* = 258	PDW ≥ 16.31%, *n* = 238	*P* value
Age (years)	52.5 ± 16.6	53.0 ± 10.6	52.4 ± 17.0	0.773
Male, *n* (%)	293 (59.1%)	94 (36.4%)	199 (83.6%)	<0.001
CCI	4.39 ± 1.71	4.28 ± 1.69	4.50 ± 1.94	0.106
Dialysis vintage (months)	85.8 (71.2, 110.3)	85.0 (70.9, 110.3)	86.1 (65.1, 99.8)	0.364
Dry weight (kg)	60.4 ± 11.1	57.6 ± 11.9	60.6 ± 13.7	0.150
BMI (kg/m^2^)	23.6 ± 5.0	21.5 ± 4.2	24.5 ± 5.3	0.049
Kt/V	1.43 (1.26, 1.64)	1.48 (1.28, 1.66)	1.36 (1.27, 1.52)	0.362
Access thrombosis, *n* (%)	115 (23.2%)	62 (24.0%)	53 (22.3%)	0.762
Access, *n* (fistula/catheter)	480/16	252/6	228/10	0.641
Systolic (mmHg)	134 ± 21	134 ± 16	140 ± 22	0.543
Diastolic (mmHg)	80 ± 15	79 ± 13	83 ± 15	0.414
Creatinine (mg/dL)	8.2 (7.5, 9.8)	6.9 (4.1, 8.3)	9.0 (8.0, 10.8)	0.005
Albumin (g/L)	43.4 ± 4.3	42.9 ± 3.3	37.0 ± 5.8	0.003
Hs-CRP (ug/mL)	2.5 (0.7, 5.3)	2.0 (0.9, 4.1)	5.8 (1.4, 7.9)	<0.001
Hemoglobin (g/L)	107.1 ± 23.8	108.6 ± 19.2	102.2 ± 24.7	0.571
Platelet counts (*x* 10^9/L)	150 ± 73.0	152 ± 66.4	144.2 ± 76.4	0.066
MPV (fL)	9.3 ± 5.3	9.2 ± 3.2	10.0 ± 7.7	0.022

PDW, platelet distribution width; CCI, Charlson comorbidity index; BMI, body mass index; hs-CRP, high-sensitive C-reactive protein; MPV, mean platelet volume.

**Table 2:** Association between PDW and all-cause and cardiovascular mortality using Cox regression models.

	Model 1		Model 2		Model 3	
	HR (95% CI)	*P*	HR (95% CI)	*P*	HR (95%CI)	*P*
*All-cause mortality*						
PDW < 16.31%	Reference					
PDW ≥ 16.31%	1.53 (1.33–5.92)	0.001	1.50 (1.21–5.11)	0.003	1.49 (1.15–6.82)	0.007

Quartiles of PDW						
Quartile 1	Reference					
Quartile 2	1.21 (1.06–5.36)	0.037	1.19 (1.05–5.41)	0.0041	1.15 (1.04–5.84)	0.045
Quartile 3	1.42 (1.09–5.17)	0.021	1.38 (1.07–5.28)	0.024	1.31 (1.06–5.53)	0.029
Quartile 4	1.69 (1.18–4.85)	<0.001	1.64 (1.21–5.02)	<0.001	1.59 (1.18–5.30)	0.001
*P* for trend	<0.001		<0.001		<0.001	

*CVD mortality*						
PDW < 16.31%	Reference					
PDW ≥ 16.31%	2.51 (1.53–3.59)	0.002	2.46 (1.62–4.01)	0.008	2.26 (1.44–3.63)	0.011

Quartiles of PDW						
Quartile 1	Reference					
Quartile 2	2.02 (1.41–3.82)	0.003	1.96 (1.37–3.97)	0.006	1.87 (1.30–4.05)	0.012
Quartile 3	2.39 (1.43–3.53)	0.001	2.31 (1.40–3.61)	0.002	2.27 (1.34–3.79)	0.003
Quartile 4	2.94 (1.59–3.58)	<0.001	2.86 (1.53–3.67)	<0.001	2.71 (1.49–3.76)	<0.001
*P* for trend	<0.001		<0.001		<0.001	

Model 1: crude analysis. Model 2: adjustment for age, sex, and CCI. Model 3: adjustment for model 2 and BMI, creatinine, albumin, hs-CRP, and MPV. PDW, platelet distribution width; CCI, Charlson comorbidity index; BMI, body mass index; hs-CRP, high-sensitive C-reactive protein; MPV, mean platelet volume.

## Data Availability

Readers can access the data underlying the findings of the study by contacting the corresponding author.
